# Medical Zoology; Being a Faithful Representation and Description of the Animals Which Belong to the Materia Medica

**Published:** 1835-07-01

**Authors:** 


*Medizinische Zoologie, oder getreue Darstellung und Beschreibung der Thiere, die in der Arzneimittellehre in Betracht kommen.* Von Dr. J. F. Brandt und Dr. J. T. C. Ratzeburg.—Berlin, 1829 und 1833.

*Medical Zoology; being a faithful Representation and Description of the Animals which belong to the Materia Medica.* By Dr. J. F. Brandt and Dr. J. T. C. Ratzeburg.—Berlin, 1829 and 1833. 2 vols. 4to. pp. 562. Sixty-three Plates.

Every animal that has the honour of furnishing any the smallest article for the medical arsenal, has a place in the work before us. Thus, the sheep, the sturgeon, the oyster, the viper, the civet cat, the burbot, the crawfish,* the blister fly, the bee, the leech, the cochineal insect, the ant,—are figured and described with German minuteness and fidelity. Some of the animals we have just mentioned, and many more, to be found in the learned volumes of Drs. Brandt and Ratzeburg, no longer contribute to the success of British practice, and are probably going out of fashion on the continent: yet we are not sorry to see them in such a work as this,—rich with the spoils of time—a work of reference for the professor, not a manual for the student, and to be consulted, rather than to be read. We strongly recommend this copious treatise to those who, unhampered by the *res angusta domi*, aspire to a complete medical library, as well as to every lecturer on zoology or materia medica.

* Every oyster-eater, that is to say, every one (with a few unhappy exceptions) knows that oysters are in season during the months with an R. Our authors inform us, that crawfish are best in the months without an R, and back their dictum with the following line, which appears to be a verse much the worse for wear.

Mensis in quo non est R. Tu debes edere cancer.

Perhaps it ought to be

Per menses sine R tu debes edere cancrum.

A very decent line for the *media vel infima Latinitas*, with only one false quantity in it.

